# Caring for Africa’s sickle cell children: will we rise to the challenge?

**DOI:** 10.1186/s12916-020-01557-2

**Published:** 2020-04-28

**Authors:** Assaf P. Oron, Dennis L. Chao, Echezona E. Ezeanolue, Loveth N. Ezenwa, Frédéric B. Piel, Osifo Telison Ojogun, Sophie Uyoga, Thomas N. Williams, Obiageli E. Nnodu

**Affiliations:** 1 Maternal, Newborn and Child Health, Institute for Disease Modeling, Bellevue, WA USA; 2 Healthy Sunrise Foundation, Las Vegas, NV USA; 3 College of Medicine, University of Nigeria, Nsukka, Nigeria; 4 National Population Commission, Abuja, Nigeria; 5 Department of Epidemiology & Biostatistics, School of Public Health, Faculty of Medicine, Imperial College London, London, UK; 6 KEMRI-Wellcome Trust Research Programme, PO Box 230, Kilifi, Kenya; 7 Centre of Excellence for Sickle Cell Disease Research & Training, University of Abuja, Abuja, Nigeria

**Keywords:** Sickle cell disease (SCD), Sickle cell anemia (SCA), Maternal newborn and child health (MNCH), Child mortality, Sustainable Development Goals (SDGs), Newborn screening

## Abstract

**Background:**

Most of the world’s sickle cell disease (SCD) burden is in Africa, where it is a major contributor to child morbidity and mortality. Despite the low cost of many preventive SCD interventions, insufficient resources have been allocated, and progress in alleviating the SCD burden has lagged behind other public-health efforts in Africa. The recent announcement of massive new funding for research into curative SCD therapies is encouraging in the long term, but over the next few decades, it is unlikely to help Africa’s SCD children substantially.

**Main discussion:**

A major barrier to progress has been the absence of large-scale early-life screening. Most SCD deaths in Africa probably occur before cases are even diagnosed. In the last few years, novel inexpensive SCD point-of-care test kits have become widely available and have been deployed successfully in African field settings. These kits could potentially enable universal early SCD screening. Other recent developments are the expansion of the pneumococcal conjugate vaccine towards near-universal coverage, and the demonstrated safety, efficacy, and increasing availability and affordability of hydroxyurea across the continent. Most elements of standard healthcare for SCD children that are already proven to work in the West, could and should now be implemented at scale in Africa. National and continental SCD research and care networks in Africa have also made substantial progress, assembling care guidelines and enabling the deployment and scale-up of SCD public-health systems. Substantial logistical, cultural, and awareness barriers remain, but with sufficient financial and political will, similar barriers have already been overcome in efforts to control other diseases in Africa.

**Conclusion and recommendations:**

Despite remaining challenges, several high-SCD-burden African countries have the political will and infrastructure for the rapid implementation and scale-up of comprehensive SCD childcare programs. A globally funded effort starting with these countries and expanding elsewhere in Africa and to other high-burden countries, including India, could transform the lives of SCD children worldwide and help countries to attain their Sustainable Development Goals. This endeavor would also require ongoing research focused on the unique needs and challenges of SCD patients, and children in particular, in regions of high prevalence.

## Background

Sickle cell disease (SCD) is caused by mutations in the β-globin gene that lead to the production of abnormal forms of the β-subunit of hemoglobin. It is the commonest life-threatening genetic disorder among people of African heritage and is also common in populations hailing from the Indian subcontinent, the Mediterranean basin, and the Middle East [1]. The majority of people with SCD suffer from the most severe form of the disease—the homozygous HbSS genotype—which is also known as sickle cell anemia (SCA). An estimated 300,000–400,000 babies with SCA are born every year, about three quarters of them across a geographical band in Africa stretching from Senegal to Madagascar, mirroring the continent’s malaria endemicity [2]. In much of this region, over 1% of all newborns have SCA. Nigeria alone is home to 25–35% of global SCD births.

In a recent review of cross-sectional population surveys and cohort studies of SCD in Africa, it was estimated that between 50 and 90% of SCA children died before age 5 years [2]. This extrapolates to 150,000–300,000 annual SCA child deaths, potentially accounting for 5–10% of the region’s total child mortality [3–5]. Many surviving children with SCA, as well as tens of thousands of additional children born in Africa every year with other forms of SCD [6], experience significant morbidity in comparison to healthy children [7, 8].

By contrast, in high-income and some middle-income countries such as Jamaica, survival among children with SCD is approaching parity with the broader population. These children also reach adulthood healthier, thanks to a public-health combination of newborn screening, preventive care, and active clinical management [9, 10]. Implementation in Africa has lagged considerably and is limited geographically. Despite SCD’s substantial health burden in Africa, there is no coherent funding effort to address it on the scale of initiatives such as the Gavi vaccine alliance or the Global Fund for malaria and HIV. Moreover, most high-burden African countries have no budgetary allocation for SCD prevention and control [11].

Recently, the US National Institutes of Health and the Gates Foundation announced a large joint allocation of funds towards the development of gene-based cures for SCD and HIV [12]. This encouraging and welcome announcement will doubtless help raise the public profile of SCD. However, to date, curative solutions available in the West have only played a very minor role in the public-health achievements described, above and are still considered a high-risk option of last resort [13, 14]. Even fulfilling this relatively modest role in Africa would require massive technological infrastructure investments. The additional cost and complexity of emerging gene-based curative solutions (the earliest of which are now only in phase I clinical trials in the West) mean that it could take several decades before such treatments reach Africa on a scale that matches SCD burden.

Meanwhile, in recent years, great strides have been made in public-health preventive and management solutions for SCD in Africa. If local and global resources could be mobilized on a scale that has already been demonstrated as feasible for other diseases, the prognosis for Africa’s SCD children can be improved dramatically within just a few years. Here, we examine the current state and outlook of public-health solutions for SCD children in Africa with an emphasis on recent progress, discuss the main barriers, and suggest some next steps. Detailed descriptions of SCD and its genetics and clinical presentation have been covered by recent reviews [4, 15, 16] and will not be repeated here.

## Main discussion

### The urgency of early screening

Children with SCD have high levels of fetal hemoglobin (HbF) in early life, rendering them generally healthy during their first few months. Once HbF production declines, their health begins to deteriorate when they might present with any of a number of conditions including anemia, painful crises, strokes, hand-foot syndrome, splenic sequestration, bacteremia, or pneumonia [14, 17]. The severity and timing of such presentations vary greatly, making early symptom-based SCD diagnosis extremely difficult.

The vast majority of Africa’s SCD children are not diagnosed before the second year of life or even later [7, 18]. If they die before they are diagnosed with SCD, death is often attributed to other causes, making SCD “an invisible killer of children” [19, 20]. Delayed diagnosis can also mean that the provision of vital education and treatment is delayed, and that children suffer escalating morbidity and irreversible end-organ damage. Their ordeals constitute a substantial yet invisible part of the general population’s child morbidity. For example, in a multihospital study conducted in Kinshasa, that included diagnosed cases a priori, 5% of children with severe anemia had undiagnosed SCA [21]. Similarly, in the 2018 National Demographic Health Survey in Nigeria, which included testing for SCD, 10% of the children aged 6 months to 5 years who were found to have severe anemia were also shown to have SCA [22]. Finally, in an 11-year chart review of stroke cases at Senegal’s only pediatric hospital, children with SCD, the majority of them undiagnosed with mean onset age of 6 years, accounted for 38% of all cases [23]. Senegal’s estimated SCD birth prevalence is 0.5–0.6% [1]. Even in high-resource countries such as Germany, the lack of universal early screening places SCD children at unacceptably high risk. Most children in a recently established national SCD registry there were discovered after age 1 year, usually by presenting with symptoms [24].

### The promise of new screening methods

Definitive SCD diagnosis requires identifying the hemoglobin type or the genotype directly. In Africa until recently, such tests have only been available in major laboratories. Despite recent progress, only a small fraction of Africa’s SCD children undergo screening in early infancy [4]. Over the past decade, several laboratory-based screening initiatives that seemed promising at first [19] have stalled or ended once external funding had dried up [4, 25].

One way out of this impasse is the use of simple, affordable point-of-care tests (POCTs). The crucial role of universal early screening and the potential of POCT methods for SCD in Africa have been noted and discussed in recent years [25–27]. Since then, progress has accelerated with at least two POCT products—SickleScan™ (BioMedomics, Morrisville, NC, USA) and HemoTypeSC™ (Silver Lake Research, Azusa, CA, USA)—having been tested in relatively large numbers under field conditions in Africa and having shown high sensitivity and specificity [28–30]. Both products are easy to use, require no power or additional materials, can be produced at high volume and low cost, and are capable of screening for SCA and other major SCD-associated genotypes in a few minutes.

In Nigeria, Nnodu et al. screened a sample of 1121 infants across more than a dozen states using HemoTypeSC™, demonstrating that POCTs can be deployed at Expanded Program for Immunization (EPI) clinics, potentially reaching the vast majority of Africa’s infants [28]. Over 80% of children born in sub-Saharan Africa (and over 90% in India) are vaccinated via EPI during the first 2 months of life [31].

Point-of-care tests can identify the two most common sickle hemoglobin types (S and C), but do not have the full diagnostic range of gold-standard methods. Therefore, they are best suited for screening many millions of infants born across vast geographies, most of them in hard-to-access rural areas. In the long run, a POCT system might be supplanted by laboratory-based newborn screening for multiple conditions, but this would require ubiquitous access to well-equipped laboratories. By contrast, EPI-integrated screening using POCTs shortly after birth could be deployed immediately in primary healthcare facilities and with minimal training.

Although not obviating the need for early-life screening, a complementary option is maternal screening at antenatal clinic (ANC) visits. Children born to mothers who do not carry the sickle cell mutation cannot have SCD. With average fertility of 5+ children per mother in West and Central Africa [32], ANC-based screening could reduce the number of valuable direct infant POCTs required by up to 70–80%. This would not result in potential SCD cases being missed, since it would only be necessary to screen each mother once and not at every pregnancy. The vast majority of African women have at least one ANC visit during pregnancy [33]. ANC screening can also help in discovering potential undiagnosed women with SCD who would be at greater risk around childbirth [34] and in informing women whether they possess the sickle cell trait. To realize these efficiencies in infant screening, data from ANC and EPI need to be integrated, and mothers well-informed.

### Promoting a proven public-health package

**Fig. 1:**
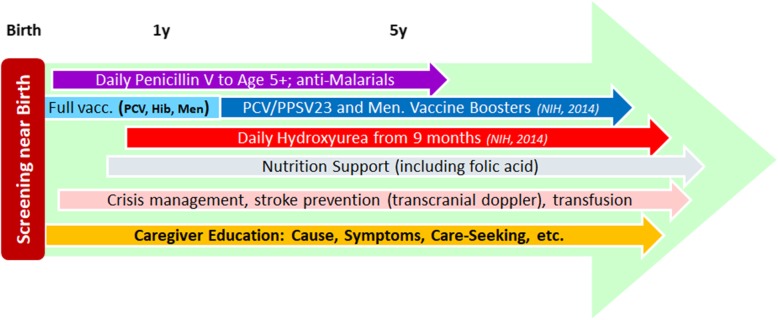
Schematic visualization of the standard public-health package to protect children with SCD

The standard public-health care package for SCD children, developed in middle- and high-income countries over the past 50–60 years, consists of several elements (Fig. 1):

1. Screening in early infancy is paramount, as discussed above.
2. Penicillin V prophylaxis during early childhood was the first preventive SCD intervention introduced in the West, with dramatic mortality reduction [35]. Some organizations now recommend continuing well beyond age 5 in high-burden, limited-resource areas [36]. Penicillin V availability and price vary across Africa [37], but it has long been a WHO Essential Medicine [38], and its global reference price is a few US cents per daily dose [39]. Therefore, both supply and cost can be stabilized. For allergic children, macrolides are indicated; they are also inexpensive and widely available.
3. In addition, SCD patients are at increased risk of malaria exacerbations due to anemia and a weakened immune system. Antimalarial prophylaxis is indicated, is widely available, and reduces the frequency of clinical episodes [40].
4. Pneumococcal conjugate vaccine (PCV) and *Haemophilus influenza* B (Hib) vaccine are both delivered during early infancy under EPI and therefore affordable and widely available. Making meningococcal vaccine in endemic regions and pneumococcal boosters at older ages universally available and affordable might require SCD-specific support.
5. Hydroxyurea promotes HbF production, among other benefits, and has been demonstrated in the West to be highly effective in reducing mortality and morbidity, including anemia mitigation, among children and adults, and in improving long-term prognosis [41, 42]. Safety and efficacy in African children were demonstrated by several recent trials [43–45] and observational studies [46–48]. Hydroxyurea is also a WHO Essential Medicine; its availability is expanding, and prices are dropping. The Stroke Prevention in children with SCA in Nigeria (SPIN) trial used hydroxyurea by a Nigerian manufacturer at US $0.13 per daily dose. In late 2019, Novartis announced it will provide hydroxyurea for free to all SCD patients in Ghana [49].
6. SCD is associated with chronic protein energy and nutrient deficiencies. Nutritional supplementation including folic acid and macro- and micronutrients to mitigate these deficiencies, as well as parent education on the value of optimal nutrition and hydration in health maintenance, is considered an essential part of preventive SCD care [50].
7. Blood transfusion to mitigate sickle cell crises, as well as transcranial Doppler ultrasound to assess the risk of stroke, both require higher levels of equipment and training than available at primary centers. In high-prevalence regions, secondary centers should be given sufficient resources to provide this care.
8. Caregiver awareness and education will be discussed further below.

Currently in Africa, it is only tertiary centers that offer a substantial part of this package. Prospective African cohorts receiving it in partial form 10–20 years ago had already experienced substantial survival improvement over historical rates [51–53]. Our hope is that it will eventually be applied in full to all African children with SCD, with most interventions available at the primary and secondary levels.

Although funders and governments have so far been reluctant to support such an expansion, the evidence suggests substantial economic benefits to society. Kuznik et al. [54] conducted a cost-effectiveness analysis of newborn screening and prevention in 43 sub-Saharan African countries. They found that at an SCD birth prevalence of more than 0.2–0.3%, screening and preventive care was highly cost-effective, costing on average < $200 per disability-adjusted life year (DALY). They identified 34 African countries meeting this threshold, despite considering only SCA births rather than any SCD and assuming no morbidity DALY benefits. They assumed screening costs of $10/sample using isoelectric focusing (IEF), the least expensive laboratory method, and did not include hydroxyurea cost and impact. Given recent progress, in particular in the development of POCTs and the accessibility of hydroxyurea, it seems plausible that a scaled-up screening and care package would be cost-effective even at a birth prevalence of 0.1% or less. In regions of ~ 1% prevalence, it is among the most cost-effective, under-utilized child health interventions available.

### Mapping SCD burden patterns using surveys

SCD birth prevalence is known to vary substantially over relatively short distances and between ethnic groups [55]. The best available global SCD prevalence maps published in 2013 [1, 6] used data from field studies conducted over six decades, with variable diagnostic methods. Recent methodological developments, combined with the inclusion of data from surveys conducted since 2010, could help to update and improve these estimates.

Nigeria’s 2018 Demographic and Health Survey (DHS) was the first-ever DHS to include SCD screening. A nationally representative sample of over 11,000 children aged 6 to 59 months was screened on location using SickleScan™ [22]. The scale and national coverage of this survey allows to identify SCA prevalence of > 1% in northeastern Nigeria, where it was previously estimated as < 0.5% based on limited older data.

An alternative method to obtain subnational information quickly has been pioneered in Uganda. Using IEF, Ndeezi et al. screened nearly 100,000 dried blood samples from national HIV child screening, producing Africa’s highest-resolution SCA prevalence map, and finding large variations: from 1–1.5% across most of the center and northeast to < 0.1% in the far southwest [56]. Older studies had suggested this general pattern, but the recent work provides far greater precision and detail. This was later followed up with targeted screening in higher-prevalence regions [57]. Similar work was carried out in northwest Tanzania, confirming an SCA prevalence there of > 1%, substantially higher than the national average [58].

### Countering stigma and misconceptions

One of the most challenging, yet essential parts of the public-health package is not a medical intervention and has received little attention thus far. Its impact was demonstrated in a recent analysis of a prospective birth cohort in Kilifi, Kenya. Infants were screened for SCA in the first year of life and were subsequently followed until the age of 5 years regardless of genotype. Roughly half of caregivers whose babies tested positive for SCA consented to enroll them into a care regimen. Their children suffered 29 deaths per 1000 person-years. SCA children whose caregivers declined enrollment suffered 104 deaths per 1000 person-years, close to the estimated 50–90% child mortality rate, despite access to modern emergency care at the Kilifi hospital. Non-enrolled SCA children were < 0.4% of the entire birth cohort, yet accounted for > 10% of under-5 deaths [53]. Even though there might be additional differences between the consenting and non-consenting groups beyond only caregiver cooperation, the fourfold difference in mortality demonstrates how societal attitudes are among the biggest obstacles to progress on SCD. Caregiver refusal to cooperate is reported from other locations across Africa [59], often at rates similar to Kilifi. Explanations vary, but are often related to the disease being less well-known, to a frightening prognosis that parents attempt to deny at first, or to misconceptions and social stigma related to the legacy of inexplicable early deaths afflicting certain families [60–63]. This is a major challenge requiring thoughtful multidisciplinary efforts and partnership with local SCD support and advocacy groups.

Knowledge and awareness gaps among clinical providers are a related challenge. A recent study from tertiary hospitals in central Nigeria found that half of doctors who routinely treat SCD patients lacked training about the use of hydroxyurea in patients with SCD and prescribed the medication 5 times less often than the other half [64].

## Conclusions and recommendations

After decades of relative neglect [19, 65], a sea change for SCD in Africa is now within reach. Strong national and continental [66] networks of SCD clinicians and researchers have been created. The references in this article demonstrate the rapidly expanding body of knowledge about SCD in Africa, thanks to locally led research. The Sickle Pan-African Research Consortium (SPARCO) has developed multilevel guidelines that can be used at all levels of healthcare, to provide standardized management for patients with SCD. SPARCO is also enrolling a large prospective multicountry SCD cohort in Tanzania, Nigeria, and Ghana, with harmonized data collection and management, and deep consideration of ethical and societal aspects [67, 68]. The Sickle Cell Support Society of Nigeria has 39 centers in all six geopolitical zones. Many tertiary healthcare centers in the country are now reasonably equipped and trained to treat SCD.

It is time for the global community to help accelerate this local mobilization and to leverage recent progress on POCTs, vaccination, and hydroxyurea, by providing appropriate financial, organizational, logistical, and research support. Particular, priorities include the following:

- ➢ The creation of a mechanism for sustainable integration of early-infancy POCT-based screening for SCD and recordkeeping in birth facilities and EPI clinics, whether via Gavi which is intimately connected to EPI, and whose broad mission is saving children’s lives, or via a similarly robust infrastructure.
- ➢ Help national health ministries to build out the requisite SCD care capacity for providing the public-health package described above, including training and supplies at the primary and secondary levels, and a full suite of advanced interventions at the tertiary level, up to and including curative solutions. This includes ensuring that the care is also affordable [18, 69].
- ➢ Support prevalence and burden surveys to help prioritize resource allocation.
- ➢ Support awareness campaigns and social research to improve caregiver awareness and cooperation, societal acceptance, and provider proficiency. In this task, it is crucial to partner with, and to help empower, patient and caregiver support and advocacy groups.
- ➢ Support implementation research to outline the most effective paths towards building these systems.
- ➢ Support the study of prospective SCD cohorts, in order to better understand patterns and modulators of severity and morbidity [70], and to tailor appropriate interventions.
- ➢ Help deploy admission screening in hospitals to identify undiagnosed patients presenting with conditions associated with untreated or undertreated SCD, such as stroke and anemia [7].
- ➢ Genetic counseling, which has complex ethical implications and depends on parent preferences and cultural heritage, may be part of the prevention toolbox. Public-health professionals should be able to provide respectful, culturally sensitive genetic screening and counseling to young adults. This counseling, already taking place in some countries, could be expanded and better supported.

These steps can be carried out first by countries ready to make the transition towards universal SCD care. The lessons learned there can then be applied elsewhere in the continent as well as in India and beyond, as the program expands. If we rise to meet this challenge, we may see SCD in Africa change soon from a pervasive killer of children to a condition managed through adulthood, preparing today’s SCD children to benefit from curative solutions once they become available.

## Data Availability

Not applicable.

## Abbreviations

ANC: Antenatal clinic
DALY: Disability-adjusted life year
DHS: Demographic and Health Survey
EPI: Expanded Program for Immunization
Gavi: The Vaccine Alliance
HbF: Fetal hemoglobin (hemoglobin F)
HbSS: Homozygous hemoglobin S genotype
HIV: Human immunodeficiency virus
IEF: Isoelectric focusing
PCV: Pneumococcal conjugate vaccine
POCT: Point-of-care test
SCA: Sickle cell anemia
SCD: Sickle cell disease
SPARCO: Sickle Pan-African Research Consortium
SPIN: Stroke Prevention In children with sickle cell anaemia in Nigeria
USA: United States of America
WHO: World Health Organization

## References

[1] Piel FB, Patil AP, Howes RE, Nyangiri OA, Gething PW (2013). Global epidemiology of sickle haemoglobin in neonates: a contemporary geostatistical model-based map and population estimates. Lancet.

[2] Piel FB, Patil AP, Howes RE, Nyangiri OA, Gething PW, Williams TN (2010). Global distribution of the sickle cell gene and geographical confirmation of the malaria hypothesis. Nat Commun.

[3] Piel FB, Hay SI, Gupta S, Weatherall DJ, Williams TN. Global burden of sickle cell anaemia in children under five, 2010-2050: modelling based on demographics, excess mortality, and interventions. PLoS Med. 2013;10(7):e1001484. 10.1371/journal.pmed.1001484 PMC3712914 23874164

[4] Kato GJ, Piel FB, Reid CD, Gaston MH, Ohene-Frempong K, Krishnamurti L, et al. Sickle cell disease. Nat Rev Dis Primer. 2018;4. 10.1038/nrdp.2018.10 29542687

[5] Simpson S (2019). Sickle cell disease: a new era. Lancet Haematol.

[6] Piel FB, Howes RE, Patil AP, Nyangiri OA, Gething PW, Bhatt S (2013). The distribution of haemoglobin C and its prevalence in newborns in Africa. Sci Rep.

[7] Macharia AW, Mochama G, Uyoga S (2018). The clinical epidemiology of sickle cell anemia in Africa. Am J Hematol.

[8] Marks LJ, Munube D, Kasirye P, Mupere E, Jin Z, LaRussa P (2018). Stroke prevalence in children with sickle cell disease in sub-Saharan Africa: a systematic review and meta-analysis. Glob Pediatr Health.

[9] Yanni E, Grosse SD, Yang Q, Olney RS (2009). Trends in pediatric sickle cell disease-related mortality in the United States, 1983-2002. J Pediatr.

[10] King L, Fraser R, Forbes M, Grindley M, Ali S, Reid M. Newborn sickle cell disease screening: the Jamaican experience (1995–2006). J Med Screen. 2016; [cited 2019 Dec 21]; Available from: https://journals.sagepub.com/doi/abs/10.1258/096914107782066185. 10.1258/096914107782066185 17925083

[11] Moeti M. Regional Committee side event on sickle cell disease [Internet]. WHO | Regional Office for Africa. 2019. Available from: https://www.afro.who.int/regional-director/speeches-messages/regional-committee-side-event-sickle-cell-disease. Cited 21 Jan 2020.

[12] NIH. NIH launches new collaboration to develop gene-based cures for sickle cell disease and HIV on global scale [Internet]. National Institutes of Health (NIH). 2019. Available from: https://www.nih.gov/news-events/news-releases/nih-launches-new-collaboration-develop-gene-based-cures-sickle-cell-disease-hiv-global-scale. Cited 16 Dec 2019.

[13] Gluckman E, Cappelli B, Bernaudin F, Labopin M, Volt F, Carreras J (2017). Sickle cell disease: an international survey of results of HLA-identical sibling hematopoietic stem cell transplantation. Blood.

[14] Hulbert ML, Shenoy S (2018). Hematopoietic stem cell transplantation for sickle cell disease: progress and challenges. Pediatr Blood Cancer.

[15] Makani J, Ofori-Acquah SF, Nnodu O, Wonkam A, Ohene-Frempong K (2013). Sickle cell disease: new opportunities and challenges in Africa. Sci World J.

[16] Piel FB, Steinberg MH, Rees DC (2017). Sickle cell disease. N Engl J Med.

[17] Ramakrishnan M, Moïsi JC, Klugman KP, Iglesias JMF, Grant LR, Mpoudi-Etame M (2010). Increased risk of invasive bacterial infections in African people with sickle-cell disease: a systematic review and meta-analysis. Lancet Infect Dis.

[18] Brown BJ, Okereke JO, Lagunju IA, Orimadegun AE, Ohaeri JU, Akinyinka OO (2010). Burden of health-care of carers of children with sickle cell disease in Nigeria. Health Soc Care Community.

[19] Ware RE (2013). Is sickle cell anemia a neglected tropical disease?. PLoS Negl Trop Dis.

[20] McGann PT, Hernandez AG, Ware RE (2017). Sickle cell anemia in sub-Saharan Africa: advancing the clinical paradigm through partnerships and research. Blood.

[21] Kadima BT, Ehungu JLG, Ngiyulu RM, Ekulu PM, Aloni MN (2015). High rate of sickle cell anaemia in Sub-Saharan Africa underlines the need to screen all children with severe anaemia for the disease. Acta Paediatr.

[22] National Population Commission - NPC/Nigeria and ICF, 2019. Nigeria Demographic and Health Survey 2018. Abuja, Nigeria, and Rockville, Maryland, USA: NPC and ICF. http://dhsprogram.com/pubs/pdf/FR359/FR359.pdf. Accessed 17 Mar 2020.

[23] Ndiaye M, Lengue F, Sagna SD, Sow AD, Fogany Y, Deme H (2018). Childhood arterial ischemic stroke in Senegal (West Africa). Arch Pédiatr.

[24] Kunz JB, Lobitz S, Grosse R, Oevermann L, Hakimeh D, Jarisch A (2019). Sickle cell disease in Germany: results from a national registry. Pediatr Blood Cancer.

[25] Hsu L, Nnodu OE, Brown BJ, Tluway F, King S, Dogara LG, et al. White paper: pathways to progress in newborn screening for sickle cell disease in sub-Saharan Africa. J Trop Dis Public Health. 2018;6(2) [cited 2019 Dec 22]. Available from: https://www.ncbi.nlm.nih.gov/pmc/articles/PMC6261323/. 10.4172/2329-891X.1000260 PMC6261323 30505949

[26] Nnodu OE (2014). Interventions for the prevention and control of sickle cell disease at primary health care centres in Gwagwalada Area Council of the Federal Capital Territory, Nigeria. Cureus.

[27] Williams TN (2015). An accurate and affordable test for the rapid diagnosis of sickle cell disease could revolutionize the outlook for affected children born in resource-limited settings. BMC Med.

[28] Nnodu O, Isa H, Nwegbu M, Ohiaeri C, Adegoke S, Chianumba R, et al. HemoTypeSC, a low-cost point-of-care testing device for sickle cell disease: promises and challenges. Blood Cells Mol Dis. 2019;78:22–28. 10.1016/j.bcmd.2019.01.007 PMC6692636 30773433

[29] Nankanja R, Kadhumbula S, Tagoola A, Geisberg M, Serrao E, Balyegyusa S. HemoTypeSC demonstrates >99% field accuracy in a sickle cell disease screening initiative in children of southeastern Uganda. Am J Hematol. 2019;94(6):E164–E166. 10.1002/ajh.25458 30859621

[30] Segbena AY, Guindo A, Buono R, Kueviakoe I, Diallo DA, Guernec G (2018). Diagnostic accuracy in field conditions of the sickle SCAN® rapid test for sickle cell disease among children and adults in two west African settings: the DREPATEST study. BMC Hematol.

[31] WHO-Unicef. WHO UNICEF coverage estimates WHO World Health Organization: Immunization, Vaccines And Biologicals. Vaccine preventable diseases Vaccines monitoring system 2019 Global Summary Reference Time Series: DTP3 [Internet]. [cited 2019 Dec 16]. Available from: https://apps.who.int/immunization_monitoring/globalsummary/timeseries/tswucoveragedtp3.html.

[32] United Nations. World Fertility Patterns 2015 [Internet]. 2016 [cited 2020 Jan 14]. Available from: https://www.un.org/en/development/desa/population/publications/pdf/fertility/world-fertility-patterns-2015.pdf.

[33] Ataguba JE-O (2018). A reassessment of global antenatal care coverage for improving maternal health using sub-Saharan Africa as a case study. PLoS One.

[34] Boafor TK, Olayemi E, Galadanci N, Hayfron-Benjamin C, Dei-Adomakoh Y, Segbefia C (2016). Pregnancy outcomes in women with sickle-cell disease in low and high income countries: a systematic review and meta-analysis. BJOG Int J Obstet Gynaecol.

[35] Petrea Cober M, Phelps SJ (2010). Penicillin prophylaxis in children with sickle cell disease. J Pediatr Pharmacol Ther.

[36] Médecins Sans Frontières (2019). Sickle cell disease - clinical guidelines [Internet].

[37] Schäfermann S, Neci R, Ndze EN, Nyaah F, Pondo VB, Heide L (2020). Availability, prices and affordability of selected antibiotics and medicines against non-communicable diseases in western Cameroon and northeast DR Congo. PLoS One.

[38] WHO (2019). World Health Organization Model List of Essential Medicines [Internet].

[39] Management Sciences for Health. International Medical Products Price Guide [Internet]. [cited 2020 Jan 9]. Available from: http://mshpriceguide.org/en/home/.

[40] Frimpong A, Thiam LG, Arko-Boham B, Owusu EDA, Adjei GO (2018). Safety and effectiveness of antimalarial therapy in sickle cell disease: a systematic review and network meta-analysis. BMC Infect Dis.

[41] NIH (2008). NIH consensus development conference: hydroxyurea treatment for sickle cell disease - panel statement [Internet].

[42] Lobo CL d C, Pinto JFC, Nascimento EM, Moura PG, Cardoso GP, Hankins JS (2013). The effect of hydroxcarbamide therapy on survival of children with sickle cell disease. Br J Haematol.

[43] Galadanci NA, Abdullahi SU, Vance LD, Tabari AM, Ali S, Belonwu R (2017). Feasibility trial for primary stroke prevention in children with sickle cell anemia in Nigeria (SPIN trial). Am J Hematol.

[44] Opoka RO, Ndugwa CM, Latham TS, Lane A, Hume HA, Kasirye P (2017). Novel use Of Hydroxyurea in an African Region with Malaria (NOHARM): a trial for children with sickle cell anemia. Blood.

[45] Tshilolo L, Tomlinson G, Williams TN, Santos B, Olupot-Olupot P, Lane A (2019). Hydroxyurea for children with sickle cell anemia in sub-Saharan Africa. N Engl J Med.

[46] Chambers TM, Kahan S, Camanda JF, Scheurer M, Airewele GE (2018). Intermittent or uneven daily administration of low-dose hydroxyurea is effective in treating children with sickle cell anemia in Angola. Pediatr Blood Cancer.

[47] Mvalo T, Topazian HM, Kamthunzi P, Chen JS, Kambalame I, Mafunga P (2019). Real-world experience using hydroxyurea in children with sickle cell disease in Lilongwe, Malawi. Pediatr Blood Cancer.

[48] Lagunju I, Brown BJ, Oyinlade AO, Asinobi A, Ibeh J, Esione A (2019). Annual stroke incidence in Nigerian children with sickle cell disease and elevated TCD velocities treated with hydroxyurea. Pediatr Blood Cancer.

[49] Press Release – Government of Ghana partner with Novartis | Ministry of Health [Internet]. [cited 2019 Dec 21]. Available from: http://www.moh.gov.gh/press-release-government-of-ghana-partner-with-novartis/.

[50] Umeakunne K, Hibbert JM (2019). Nutrition in sickle cell disease: recent insights. Nutr Diet Suppl.

[51] Rahimy MC, Gangbo A, Ahouignan G, Adjou R, Deguenon C, Goussanou S (2003). Effect of a comprehensive clinical care program on disease course in severely ill children with sickle cell anemia in a sub-Saharan African setting. Blood.

[52] Makani J, Cox SE, Soka D, Komba AN, Oruo J, Mwamtemi H (2011). Mortality in sickle cell anemia in Africa: a prospective cohort study in Tanzania. PLoS One.

[53] Uyoga S, Macharia AW, Mochamah G, Ndila CM, Nyutu G, Makale J (2019). The epidemiology of sickle cell disease in children recruited in infancy in Kilifi, Kenya: a prospective cohort study. Lancet Glob Health.

[54] Kuznik A, Habib AG, Munube D, Lamorde M (2016). Newborn screening and prophylactic interventions for sickle cell disease in 47 countries in sub-Saharan Africa: a cost-effectiveness analysis. BMC Health Serv Res.

[55] Weatherall DJ (2010). The importance of micromapping the gene frequencies for the common inherited disorders of haemoglobin. Br J Haematol.

[56] Ndeezi G, Kiyaga C, Hernandez AG, Munube D, Howard TA, Ssewanyana I (2016). Burden of sickle cell trait and disease in the Uganda Sickle Surveillance Study (US3): a cross-sectional study. Lancet Glob Health.

[57] Kiyaga C, Hernandez AG, Ssewanyana I, Schaefer BA, McElhinney KE, Ndeezi G (2019). Sickle cell screening in Uganda: high burden, human immunodeficiency virus comorbidity, and genetic modifiers. Pediatr Blood Cancer.

[58] Smart LR, Ambrose EE, Charles M, Hernandez AG, Latham TS, Hokororo A, et al. Genetic analysis in the Tanzania Sickle Surveillance Study (TS3): modifiers of sickle cell disease and identification of hemoglobin variants. Blood. 2019;134(Supplement 1):988.

[59] Nnodu OE. Interventions for the prevention and control of sickle cell disease at primary health care centres in Gwagwalada Area Council of the Federal Capital Territory, Nigeria. Cureus. 2014. Available from: http://www.cureus.com/articles/2554-interventions-for-the-prevention-and-control-of-sickle-cell-disease-at-primary-health-care-centres-in-gwagwalada-area-council-of-the-federal-capital-territory-nigeria. Cited 22 Dec 2019.

[60] Marsh VM, Kamuya DM, Mlamba AM, Williams TN, Molyneux SS (2010). Experiences with community engagement and informed consent in a genetic cohort study of severe childhood diseases in Kenya. BMC Med Ethics.

[61] Marsh VM, Kombe F, Fitzpatrick R, Williams TN, Parker M, Molyneux S. Consulting communities on feedback of genetic findings in international health research: sharing sickle cell disease and carrier information in coastal Kenya. BMC Med Ethics. 2013;14(1):41. 10.1186/1472-6939-14-41 PMC4016314 24125465

[62] Nzewi E (2001). Malevolent Ogbanje: recurrent reincarnation or sickle cell disease?. Soc Sci Med.

[63] Dennis-Antwi JA, Culley L, Hiles DR, Dyson SM (2011). ‘I can die today, I can die tomorrow’: lay perceptions of sickle cell disease in Kumasi, Ghana at a point of transition. Ethn Health.

[64] Ofakunrin A, Adekola K, Okpe E, Oguche S, Afolaranmi T, Kanhu P (2019). Level of utilization and provider-related barriers to hydroxyurea use in the treatment of sickle cell disease in Jos, Nigeria. Blood.

[65] Grosse SD, Odame I, Atrash HK, Amendah DD, Piel FB, Williams TN (2011). Sickle cell disease in Africa: a neglected cause of early childhood mortality. Am J Prev Med.

[66] SickleInAfrica Consortium | https://www.sickleinafrica.org [Internet]. [cited 2020 Jan 7]. Available from: https://www.sickleinafrica.org/.

[67] Munung NS, Nembaware V, de Vries J, Bukini D, Tluway F, Treadwell M (2019). Establishing a multi-country sickle cell disease registry in Africa: ethical considerations. Front Genet.

[68] Wonkam A, Makani J (2019). Sickle cell disease in Africa: an urgent need for longitudinal cohort studies. Lancet Glob Health.

[69] Adegoke SA, Abioye-Kuteyi EA, Orji EO (2014). The rate and cost of hospitalisation in children with sickle cell anaemia and its implications in a developing economy. Afr Health Sci.

[70] Wonkam A, Bitoungui VJN, Vorster AA, Ramesar R, Cooper RS, Tayo B (2014). Association of Variants at BCL11A and HBS1L-MYB with hemoglobin F and hospitalization rates among sickle cell patients in Cameroon. PLoS One.

